# Phylogeography of lethal male fighting in a social spider mite

**DOI:** 10.1002/ece3.4770

**Published:** 2019-01-24

**Authors:** Yukie Sato, Yoshiaki Tsuda, Hironori Sakamoto, Martijn Egas, Tetsuo Gotoh, Yutaka Saito, Yan‐Xuan Zhang, Jian‐Zhen Lin, Jung‐Tai Chao, Atsushi Mochizuki

**Affiliations:** ^1^ Sugadaira Research Station, Mountain Science Center University of Tsukuba Ueda Nagano Japan; ^2^ Institute for Agro‐Environmental Sciences National Agriculture and Food Research Organization Tsukuba Ibaraki Japan; ^3^ Laboratory of Applied Entomology and Zoology, Faculty of Agriculture Ibaraki University Ami Ibaraki Japan; ^4^ National Institute for Environmental Studies Tsukuba Ibaraki Japan; ^5^ Institute for Biodiversity and Ecosystem Dynamics University of Amsterdam Amsterdam The Netherlands; ^6^ Faculty of Economics Ryutsu Keizai University Ryugasaki Ibaraki Japan; ^7^ Institute of Plant Protection Fujian Academy of Agricultural Sciences Fuzhou China; ^8^ Research Faculty of Agriculture Hokkaido University Sapporo Kita‐ku Japan; ^9^ Division of Forest Protection Taiwan Forestry Research Institute Taipei Taiwan, ROC

**Keywords:** contest behavior, geographic variation, haplodiploidy, phylogeography, spider mite, the Japanese archipelago

## Abstract

When males fight for access to females, such conflict rarely escalates into lethal fight because the risks and costs involved, that is, severe injury or death, are too high. The social spider mite, *Stigmaeopsis miscanthi,* does exhibit lethal male fights, and this male–male aggressiveness varies among populations. To understand the evolution of lethal fighting, we investigated aggressiveness in 42 populations and phylogenetic relationships in 47 populations along the Japanese archipelago. By analysis of the male weapon morph, a proxy for aggressiveness, we confirmed the existence of a mildly aggressive (ML) form, besides the low aggression (LW) and high aggression (HG) forms reported earlier. To evaluate demographic history of these three forms, we employed the approximate Bayesian computation approach using mtCOI sequences and taking into consideration the postlast glacial expansion history of the host plant, *Miscanthus sinensis*. As results, hierarchical split models are more likely to explain the observed genetic pattern than admixture models, and the ML form in the subtropical region was considered the ancestral group. The inferred demographic history was consistent with the one reconstructed for the host plant in a previous study. The LW form was split from the ML form during the last glacial period (20,000–40,000 years BP), and subsequently, the HG form was split from the ML form at the end of or after the last glacial period (5,494–10,988 years BP). The results also suggest that the mite invaded Japan more than once, resulting in the present parapatric distribution of LW and HG forms in eastern Japan.

## 1 INTRODUCTION

In the animal kingdom, males often fight with conspecific rival males for access to females (Andersson, 1994). However, male fights rarely escalate into lethal fight, because death or severe injury in fights represents too much risk and cost for males (Maynard Smith & Price, 1973). Indeed, males often show display behavior which allows opponent males to estimate the fighting ability and thereby avoid unnecessary fights, and also show nonfighting behavior (alternative male mating tactics) such as sneaking, satellite behavior, and female mimicking (Andersson, 1994; Gross, 1996). Nevertheless, in several species, males do fight to the death (Crespi, 1988; Hamilton, 1979; Saitō, 1990; Zenner, O'Callaghan, & Griffin, 2014). Why and how such lethal male fight evolves may be explained by several adaptive mechanisms, for example, extremely high benefit that each mating event contributes to lifetime reproduction of the males (Enquist & Leimar, 1990), low encounter rate with male competitors (Innocent, West, Sanderson, Hyrkkanen, & Reece, 2011; Murray, 1987, 1989), and difference in genetic relatedness among males (Hamilton, 1979; Kapranas, Maher, & Griffin, 2016; Reinhold, 2003; Saito, 1995; Sato, Egas, Sabelis, & Mochizuki, 2013). Few studies have addressed a reconstruction of the evolutionary process, despite both approaches being important for understanding evolution of lethal fighting.

**Figure 1:**
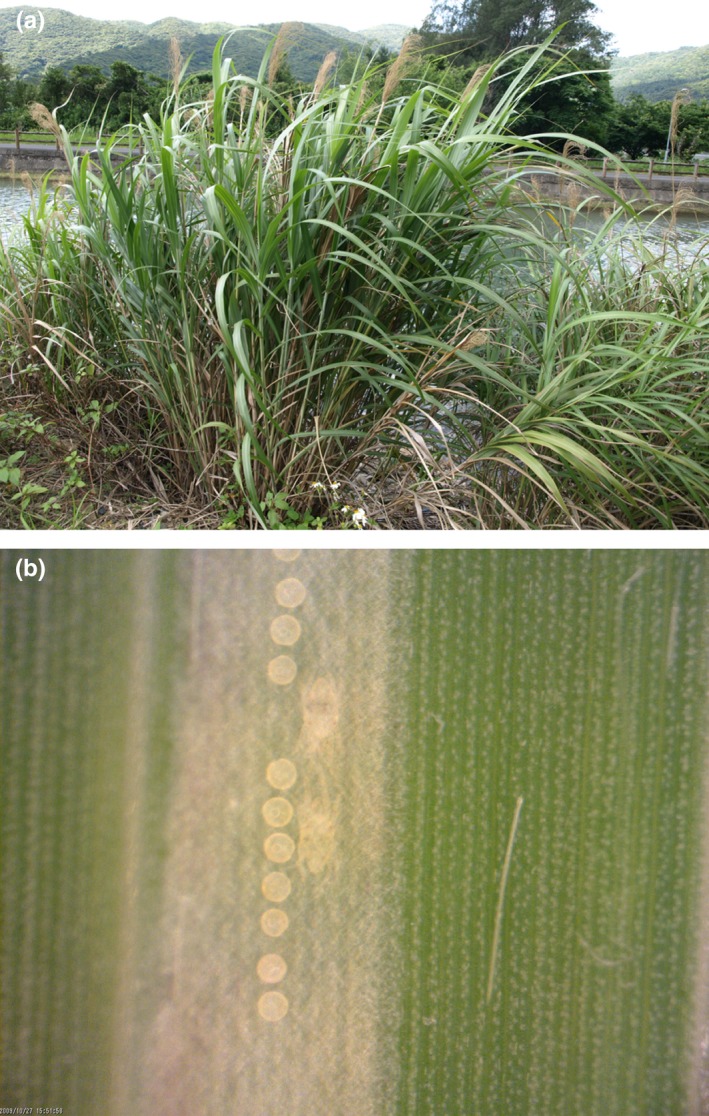
Photos of (a) a stand of Chinese silver grass (*Miscanthus sinensis*), the host plant of the social spider mite *Stigmaeopsis miscanthi*, and (b) two adult males of *S. miscanthi* in fighting position, facing each other with front legs spread out, inside a nest

*Stigmaeopsis miscanthi* (Saito) is a spider mite which infests Chinese silver grass, *Miscanthus sinensis* Andersson (Figure 1a). The mite forms colonies on the host plant by constructing woven nests on the undersurface of host plant leaves. Adult males and females counterattack intruders such as predatory mites to protect their nest mates and offspring inside the nests (Saitō, 1986; Saito, 2010). Males are aggressive not only against predators but also against other conspecific males inside the nests and even kill rival males to establish their own harem (Figure 1b). The frequency of male killing (hereafter, male–male aggressiveness) varies among populations, and male–male aggressiveness is low in colder regions, high in warmer regions, and mild in subtropical regions in Japan (Saito, 1995; Saito & Sahara, 1999; Sato, Egas, et al., 2013). Populations with low and high aggressiveness are discriminated as LW and HG forms, because of their behavioral, ecological, and morphological differences (Saito & Sahara, 1999; Saito, Sakagami, & Sahara, 2002; Yano, Saito, Chittenden, & Sato, 2011), their molecular phylogeny (Ito & Fukuda, 2009; Sakagami, Saito, Kongchuensin, & Sahara, 2009; Sakamoto et al., 2017) and also incomplete but strong reproductive isolation between them (Sato, Breeuwer, Egas, & Sabelis, 2015; Sato, Saito, & Mori, 2000a, 2000b). Recently, populations with mild male–male aggressiveness and intermediate male weapon morph were found (Sato, Egas, et al., 2013), yet their ecological and phylogenetic relationships with the LW and HG forms are unclear (Sato et al., 2018). Using a different set of sampled populations, Sakamoto et al., (2017) identified a third clade in *S. miscanthi,* which consisted of a population collected from Fuzhou, China, by phylogenetic analysis based on COI and 18S–28S genes. However, the male–male aggressiveness and the male weapon morph of this population were not determined. Together, these results suggest there are at least three clades of *S. miscanthi* differing in male–male aggressiveness. The level of male–male aggressiveness appears to be a function of average genetic relatedness among nest mates, as the geographic variation in male–male aggressiveness is related to winter harshness, which is a proxy of average genetic relatedness among nest mates via inbreeding in the mite (Saito, 1995; Sato, Egas, et al., 2013; Saito & Sahara, 1999). Although the adaptive mechanism has been addressed from the viewpoint of kin selection theory (Saito, 1995; Sato, Egas, et al., 2013; Saito & Sahara, 1999), the kin structure has not been measured directly.

**Figure 2:**
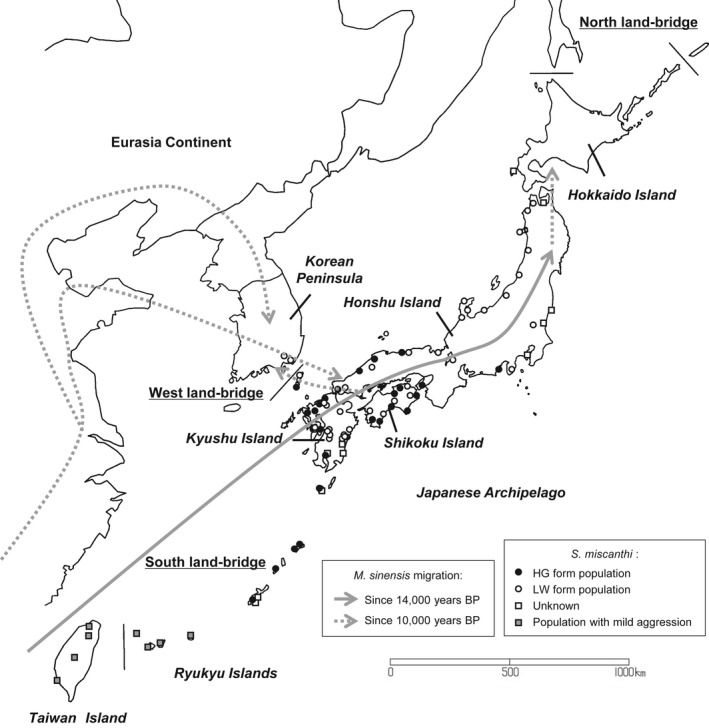
Geographic distribution of the social spider mite, *Stigmaeopsis miscanthi* and invasion route of the host plant, *Miscanthus sinensis*, during the last glacial period in the Japanese archipelago and the surrounding area. Open and filled circles show the mite populations of the low aggression (LW) and high aggression (HG) forms (Saito, 1995; Sato, Egas, et al., 2013; Saito & Sahara, 1999). Open and filled squares show the mite populations in which either of aggression or male weapon morph has not been investigated (Saito, 1995; Saito & Sahara, 1999) and the mite populations which exhibit intermediate aggressiveness between the HG and LW forms (Sato, Egas, et al., 2013). Solid and dotted gray arrows show the primary *M. sinensis* migration route into Japanese Archipelago and the secondary *M. sinensis* migration route through East China and Korea Peninsula of the host plant (Clark et al., 2014)

For the evolutionary process leading to the current geographic variation in male–male aggressiveness in the mite, several scenarios can be considered. The past expansion history of the host plant *M. sinensis* in relation to the last glacial maximum (LGM, ca. 21,000 years BP) may be especially important, since evolution in arthropod herbivores is often associated with the host plants (Janz, Nylin, & Wahlberg, 2006; Schluter, 2000; Tilmon, 2008). Population genetic studies on the host plant *M. sinensis* suggested that the northern limit of the distribution range of *M. sinensis* was probably around the coastal areas from southern Taiwan to Hainan close to the South China Sea during the LGM (Figure 2) (Clark et al., 2014). Subsequently, from 14,000 years BP, *M. sinensis* likely expanded directly from southeastern China to Japan via the south land bridge and subsequently colonized eastern China and Korea (Clark et al., 2014). The migration routes of *M. sinensis* to Korea are estimated to be not only from southeastern China, but also from Japan via the west land bridge (Clark et al., 2014). If the mite shared the colonization episode together with *M. sinensis* or followed the migration, the populations with mild aggressiveness in the subtropical region are expected to be the ancestor group, and the two forms with low and high aggressiveness emerged during its migration into the north. It is currently possible to test these several candidate scenarios of the past demographic history of species or populations using the approximate Bayesian computation (ABC) approach with genetic data (Bertorelle, Benazzo, & Mona, 2010). Thus, applying the ABC approach to the three groups of *S. miscanthi* has great potential to shed light on our understanding of the evolutionary history of population demography of the mites and thereby on the evolutionary process of divergent male–male aggressiveness.

The aim of this study is to understand the evolutionary process of lethal male fighting in the social spider mite* S. miscanthi*. In this study, we first describe the male weapon morph in the mite populations distributed in the area covering the west and south land bridges. Using male weapon morph as proxy, we investigate whether the mite populations can be categorized into three distinct forms: low, high, and mild aggression forms (Sato, Sabelis, & Mochizuki, 2013; Sato, Saito, & Chittenden, 2008; Sato, Saito, & Mori, 2000a, 2000b). Second, we determine phylogenetic relationships of the populations by analyzing sequences of the nuclear parasodium channel gene and the mitochondrial DNA cytochrome oxidase I (mtCOI). Finally, we test the hypothesis of migration history of the mite by inferring the past demographic history of the three forms using the ABC approach.

## 2 MATERIALS AND METHODS

### 2.1 Host plants and mites

Chinese silver grass, *M. sinensis*, was collected from Tsukuba (Ibaraki Prefecture, Japan) in March 2008. Its roots were pulled apart, and the resulting clones were grown in a greenhouse to be used for mite rearing and experiments.

The social spider mite, *S. miscanthi*, was collected from 41 sites: the Korean Peninsula (two sites), the Kyushu region (18 sites), the Ryukyu Islands (17 sites including the Northern Ryukyus [two sites], the Central Ryukyus [six sites], and the Southern Ryukyus [nine sites]), and Taiwan Island (four sites). Mite collection was carried out from December 2008 to July 2009. The collected mites were reared on the host plant leaves for 4–6 months under controlled conditions at 18–25°C, 60%–80% relative humidity, and 15:9 hr light:dark (L:D). These mite cultures were used for the measurement of male weapon morph. Besides these populations, male specimens immersed in MA 80 fluid (Saito, Osakabe, Sakagami, & Yasui, 1993) of *S. miscanthi* collected from Fujian Province in China in July 2011 were used in the measurement.

For the phylogenetic analysis, a number of collected female mites of each population were kept in 100% ethanol or acetone in a microtube. Female specimens of *S. miscanthi* immersed in 100% ethanol or acetone collected from Fujian Province in China (two sites), Taiwan (two sites), the Central Ryukyus (one site), and the Kyushu region (one site), and the specimens of the closely related species, *Stigmaeopsis longus* (Saito) mites collected from Fukuoka in Kyushu Island and Tsukuba in the mainland of Japan (two sites), were also used for the phylogenetic analysis.

### 2.2 Male weapon morph

Males use the first pair of legs in the male fights. The relative length of leg I to leg III (leg I/leg III) is significantly correlated with male–male aggressiveness defined as the probability of one of two males dying within a period of 5 days (Saito, 1995; Sato, Egas, et al., 2013). Therefore, we used the relative length of leg I to evaluate male–male aggressiveness in the populations.

We used 42 *S. miscanthi* populations in the measurements of relative length of leg I. From each mite culture and specimen immersed in MA liquid, 8–22 males were arbitrarily collected. These males were prepared separately as slide specimens using Hoyer's solution. The specimens were dried by pressing them with a 10‐g weight at 45°C for 3 days to flatten the body for accurate measurement. The lengths of the tibia, tarsus, genu, and femur on legs I and III were measured using a photomicrograph and image processing software (Image J ver. 1.41; National Institutes of Health, Bethesda, MD, USA). The lengths of 19 out of 42 mite populations were obtained from previous studies (Sato, Egas, et al., 2013), since the measurements were carried out in the same way and in the same period. The relative length of leg I to leg III (leg I/leg III) was calculated from the totals of the four leg segment lengths. The relative lengths of male leg I (leg I/leg III) were log‐transformed, and the population average value of the transformed data was used in the hierarchical cluster analysis using Euclidean distance and Ward's method (Murtagh & Legendre, 2014) with statistic free software R (R Core Team, 2015).

### 2.3 DNA preparation and sequencing

For the phylogenetic analyses, we used 47 populations of *S. miscanthi* collected from Korea, Japan, Taiwan, and China and two populations of *Stigmaeopsis longus* collected from Fukuoka Prefecture and Ibaraki Prefecture, Japan. DNA was extracted from a single female mite from each population, which was stored in 100% ethanol or acetone in a microtube, by using Wizard Genomic DNA Purification Kit (Promega, Madison, WI, USA), PrepMan Ultra Sample Preparation Reagent (Applied Biosystems, Foster City, CA, USA), or QIAamp DNA micro kit (Qiagen K. K., Tokyo, Japan). To amplify the fragment of parasodium channel gene, polymerase chain reaction (PCR) is carried out using primers given in Supporting Information Table S1 (Sato et al., 2018) in a 20 μl reaction mixture containing 1 μl of DNA sample, 2 μl of 10× *Ex Taq* buffer (20 mM mg^2+^ plus; Takara Bio Inc., Otsu, Shiga, Japan), 0.2 μl of *TaKaRa* *Ex Taq* (5 U/μl; Takara Bio Inc.), 1.6 μl of dNTP mix (2.5 mM each; Takara Bio Inc.), 1 μl of each primer (10 pmol/ul each), and 13.2 μl of ddH_2_O. PCR cycling conditions were 2 min at 94°C, followed by 38 cycles of 30 s at 94°C, 30 s at 53°C, and 1 min at 72°C, and a final extension at 72°C for 10 min. To amplify the fragment of mtCOI gene, PCR is carried out using primers given in Supporting Information Table S1 (Matsuda, Morishita, Hinomoto, & Gotoh, 2014) in a 10 μl reaction mixture containing 1 μl of DNA sample, 1 μl of 10× *Ex Taq* buffer (20 mM mg^2+^ plus; Takara Bio Inc.), 0.05 μl of *TaKaRa* *Ex Taq* (5 U/μl; Takara Bio Inc.), 0.8 μl of dNTP mix (2.5 mM each; Takara Bio Inc.), 0.5 μl of each primer (10 pmol/μl each), and 6.35 μl of ddH_2_O. PCR cycling conditions were 4 min at 94°C, followed by 35 cycles of 1 min at 94°C, 1 min at 45°C, and 1.5 min at 72°C, and a final extension at 72°C for 10 min. In some samples, the fragment was not amplified. Therefore, in the samples, PCR is carried out using another primers in Table 1 (Sato et al., 2018) in a 50 μl reaction mixture containing 1 μl of DNA sample, 25 μl of Premix Ex Taq (1.25 U/25 μl; Takara Bio Inc.), and 24 μl of ddH_2_O, and PCR conditions were 1 min at 94°C, followed by 35 cycles of 10 s at 98°C, 30 s at 50°C, and 1 min at 72°C without a final extension. PCR products were purified using QIA quick PCR Purification Kit (QIAGEN) or ExoSAP‐IT PCR Product Cleanup (Affymetrix Inc., Cleveland, OH, USA), and the purified products were sequenced using ABI BigDye Terminator ver. 3 Cycle Sequencing Kit (Applied Biosystems) and ABI3130xlGenetic Analyzer (Applied Biosystems). A part of the samples was sequenced by an external service (FASMAC Co., Ltd., Atsugi, Kanagawa, Japan). The sequences data of 12 out of 47 *S. miscanthi* populations and one of two *Stigmaeopsis longus* populations were obtained from previous studies (Sato et al., 2018) (GenBank accession numbers: MH015200–MH015213, MH029789–MH029801), since the DNA preparation and sequences were carried out in the same way, the same populations and the same period. The sequence data of 35 *S. miscanthi* populations and one *Stigmaeopsis longus* population obtained in this study have been submitted to GenBank database under accession number MH203500–MH203571.

**Table 1:** Posterior probability of each scenario and its 95% hyper probability density (HPD) based on the logistic estimate by DIYABC

Scenario	Posterior probability	95% HPD:lower–upper
1	0.0822	0.0614–0.1030
2	0.4631	0.4397–0.4865
3	0.0161	0.0000–0.0380
4	0.0224	0.0006–0.0442
5	0.1183	0.0983–0.1383
6	0.0025	0.0000–0.0248
7	0.0434	0.0223–0.0645
8	0.0699	0.0492–0.0907
9	0.1820	0.1522–0.2119

### 2.4 Phylogenetic analyses

Obtained sequences of parasodium channel gene and of mtCOI were aligned using CLUSTAL W (codons) and CLUSTAL W (DNA), respectively (Thompson, Higgins, & Gibson, 1994) with default settings in MEGA6 (Tamura, Stecher, Peterson, Filipski, & Kumar, 2013). A maximum‐likelihood tree of each region was constructed with MEGA 6 (Tamura et al., 2013). The Hasegawa–Kishino–Yano model with and without gamma‐distributed rates was used as the substitution model for the maximum‐likelihood trees of parasodium channel gene region and of mtCOI region, respectively, according to Bayesian information criteria (BICs) in ML fits of 24 different nucleotide substitution models. Reliability of trees was evaluated by the bootstrap test (*N* = 1,000). Since most of the nodes in the maximum‐likelihood tree were poorly supported by bootstrapping (see Supporting Information Figures S1 and S2), neighbor‐net (Bryant & Moulton, 2004) was generated using the software SplitsTree4 (Huson & Bryant, 2006) and employed in this paper to show their phylogenetic relationship.

### 2.5 Inference of past demographic history of three forms

**Figure 3:**
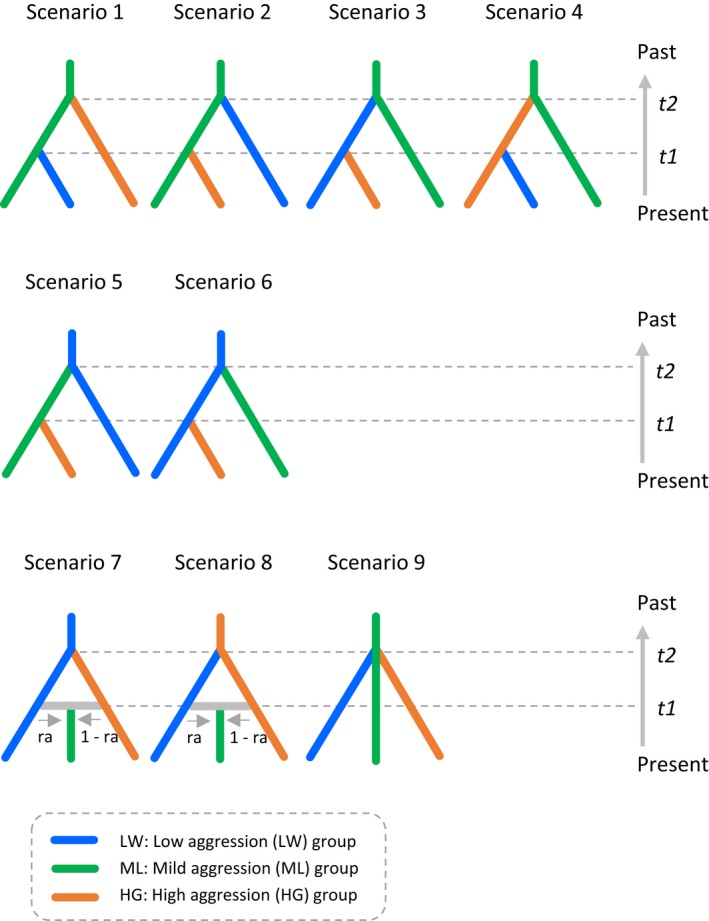
Nine demographic scenarios of the three forms in *Stigmaeopsis miscanthi* tested by DIYABC. Based on male weapon morph and phylogenetic relationship, 42 populations of the mite were pooled into three form groups: LW, ML, and HG groups. Scenarios 1–4 are based on hypothesis 1; ML form in subtropical region is the ancestor group, and the other two forms with low and high aggressiveness emerged while its migration into the north. Scenarios 5–6 are based on hypothesis 2: LW form showing low aggressiveness is the ancestor group; then, the aggressiveness became stronger gradually resulting in ML and HG forms. Scenarios 7–8 are based on hypothesis 3; ML form with intermediate aggressiveness was generated by admixture of LW and HG forms with low and high aggressiveness. Scenario 9 assumes that the three forms were split at the same time. Blue, green, and orange represent LW, ML, and HG forms, and *t*# represents timescale measured in the number of generations. For the prior distributions of the parameters used in DIYABC, see Supporting Information Table S2. For present geographic relationship of the three forms, see Figure 4c

To infer how the three forms were generated by past demographic history along the temporal scale based on the ABC approach, we examined nine simple scenarios by using mtCOI sequences of 10 LW populations, 13 mild aggressive populations (except for F1 and F2, hereafter, we call these ML populations or ML form, because of the results in cluster analysis using the male weapon morph), and 18 HG populations and the software DIYABC ver. 2.0 (Cornuet et al., 2014). The examined scenarios are as follows and shown in Figure 3.

*Scenarios 1–4: Hierarchical split models based on the hypothesis (Hypothesis 1)*. In these scenarios, the ML form is assumed to be the common ancestor of the other two forms. In scenarios 1 and 2, both the LW and HG forms derived from the ML form, but at different times (*t*1 or *t*2). In scenarios 3 and 4, either the LW or the HG form derived from the ML form at *t*2, and then the third form derived from the LW or HG form at *t*1.

*Scenarios 5–6: Other hierarchical split models (Hypothesis 2)*. These scenarios assume that the LW form is the ancestor group. This is worth considering as alternative scenarios, because the mite species derived from *Stigmaeopsis longus*, which exhibits no male killing behavior, by a host shift from Sasa bamboo to the Chinese silver grass (Ito & Fukuda, 2009; Sakagami et al., 2009; Sakamoto et al., 2017). In scenario 5, the ML form derived from the LW form at *t*2, and the HG form derived from the ML form at *t*1. In scenario 6, both the ML and HG forms derived from the LW form, at *t*2 and *t*1, respectively.

*Scenario 7–8: Isolation with admixture models (Hypothesis 3)*. In these scenarios, the ML form is assumed to be generated by admixture between the LW and HG forms at time *t*1, that is, assuming that hybrids of HG and LW forms display intermediate levels of lethal male fighting. The LW and HG forms split at time *t*2; ancestor was the LW form in scenario 7 and was the HG form in scenario 8.

*Scenario 9: Simple split model (Hypothesis 3).* The three forms derive from one split at time *t*2.

In all scenarios, *t*# represents the timescale measured in the number of generations. The prior distribution of effective population size of the form group (hereafter, N‐form) and maximum values of timescales were extended from the default values to obtain better fit of the model, according to the pilot runs of DIYABC (Supporting Information Table S2). Except for these, default values of the priors were used for other parameters (Supporting Information Table S2). The Hasegawa–Kishino–Yano (Hasegawa, Kishino, & Yano, 1985) model was employed as mutation model of mtCOI. The number of haplotypes, the number of segregating sites, the mean of pairwise differences, the variance of pairwise differences, Tajima's *D*, the number of private segregating sites, and the mean of numbers of the rarest nucleotide at segregating sites were used as summary statistics for each of the three form groups. The number of haplotypes, the number of segregating sites, the mean of pairwise differences, and *F*_ST_ (Hudson, Slatkin, & Maddison, 1992) were used as the summary statistics for each of the form group pairs. A million simulations were run for each scenario. After all the simulations had been run, the most likely scenario was determined by comparing the posterior probabilities using the logistic regression method. The goodness of fit of the scenario was assessed by the option “model checking” with principal component analysis (PCA) in DIYABC, which measures the discrepancy between model and real data.

## 3 RESULTS

### 3.1 Male weapon morph

**Figure 4:**
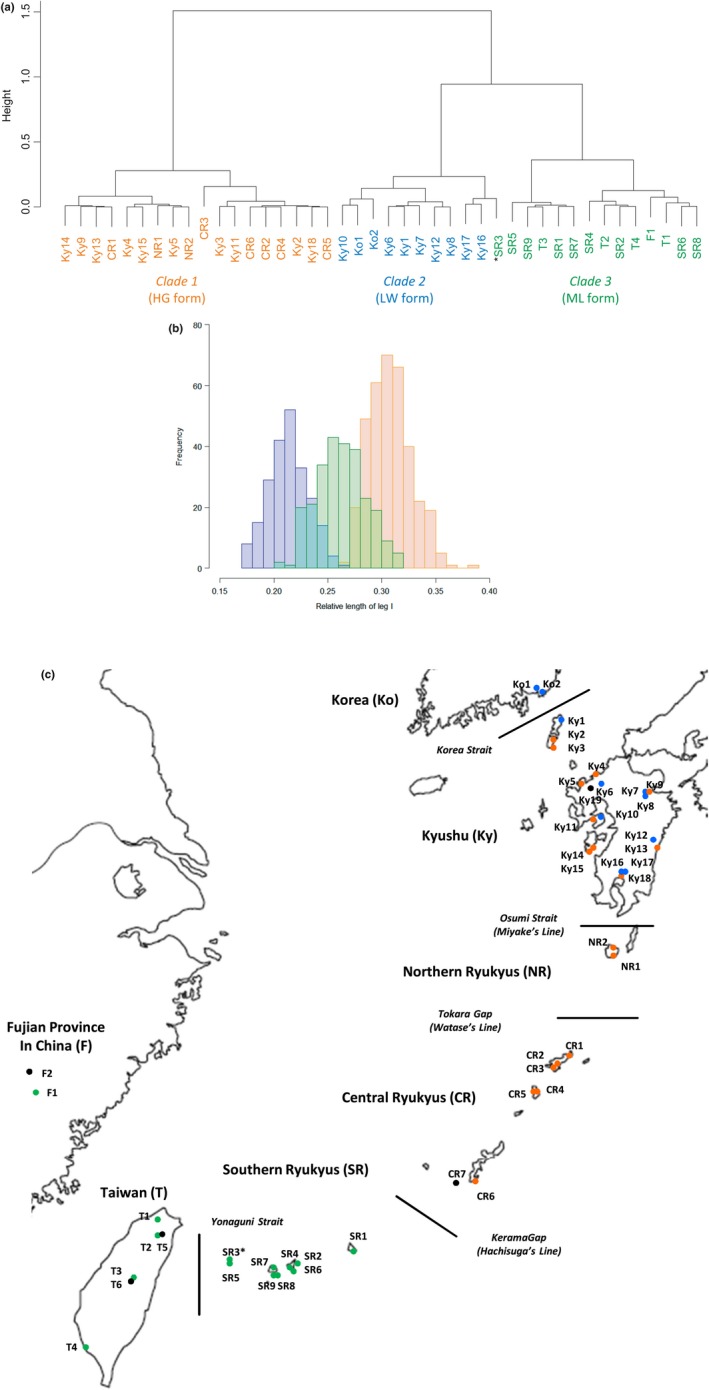
(a) Dendrogram created by hierarchical cluster analysis using average relative length of male leg I from 42 populations of *Stigmaeopsis miscanthi*, (b) histogram of relative length of male leg I of 834 individuals from the populations categorized into the three forms by the cluster analysis, and (c) localities of the populations including unknown‐form populations (indicated as black dots) used in phylogenetic analyses. The populations are grouped into seven regions based on current faunal characteristics and geographic features and numbered in order of north latitude in each region. The average relative length of male leg I used in the analysis is shown in Supporting Information Table S3. Orange, blue, and green show populations categorized as HG form, LW form, and ML form, respectively, and shaded colors in the histogram (b) show the overlaps between ML (green) and LW (blue) and between ML (green) and HG (orange). SR3 marked by asterisk was categorized as LW form in the dendrogram (clade 2 in a); however, because of the average value (Supporting Information Table S3) and the phylogenetic relationship (Figure 5), SR3 was deemed as ML form

Cluster analysis based on the male weapon morph (relative length of leg I of the males) showed three clades (Figure 4a). Clade 1 consists of populations with the longest relative length of leg I distributed from Kyushu to the Central Ryukyus, corresponding to the HG form (Figure 4b,c). Clade 2 mainly consists of populations with the shortest relative length of leg I distributed from Korea to Kyushu, corresponding to the LW form (Figure 4b,c). Clade 3 consists of populations with intermediate relative length of leg I distributed from the South Ryukyus and Taiwan to Fujian Province in China, which confirms that the third, ML form can also be identified by morphology (Figure 4b,c). One population, SR3 was categorized in clade 2 from the South Ryukyus in the cluster analysis (Figure 4a). However, the average value of relative length of leg I (0.234; Supporting Information Table S3) was bordering between clade 2 (LW populations) and the clade 3 (ML populations). Besides, in the following phylogenetic analyses, SR3 belonged to the clade of ML form. Therefore, SR3 is deemed to be an ML population, and the results suggest that, besides male weapon morph, other morphological characters are necessary to identify them more strictly.

### 3.2 Phylogenetic analyses

#### 3.2.1 Nuclear parasodium channel gene

**Figure 5:**
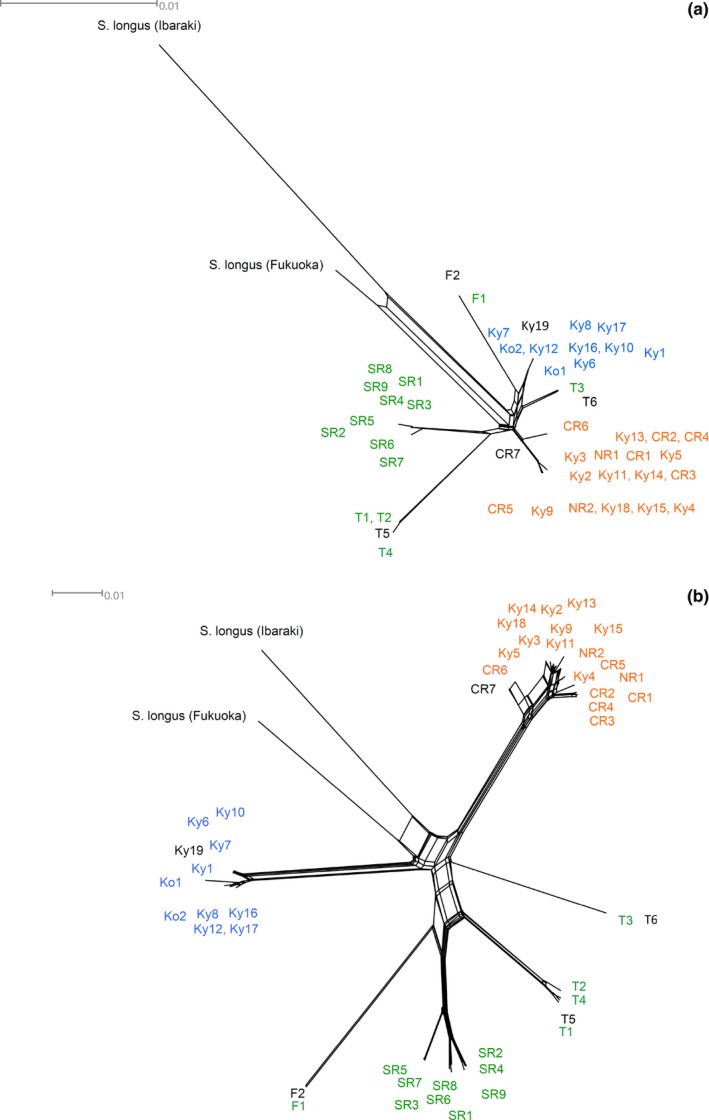
Phylogenetic networks of 47 populations of *Stigmaeopsis miscanthi* and two populations of *Stigmaeopsis longus* based on (a) parasodium channel gene and (b) mtCOI. Blue, green, and orange represent LW, ML, and HG populations categorized by a cluster analysis (Figure 4a), and black represents unknown‐form populations of *S. miscanthi* and *Stigmaeopsis longus *populations. For the localities of *S. miscanthi *populations, see Figure 4c

After alignment, the fragment had 1,310 nucleotide sites including 57 parsimony informative sites (44 parsimony informative sites when *Stigmaeopsis longus* was excluded). In the phylogenetic network based on this sequence, HG populations and LW populations (clades 1 and 2 in Figure 4a) each formed a single clade (Figure 5a). However, ML populations (clade 3 in Figure 4a) are split into four clades (Figure 5a): two populations from Fujian Province in China, four populations from Taiwan, nine populations from the Southern Ryukyus, and two populations from mountainous area in Taiwan (>1,000 m above sea level) (Figure 5a). The results were consistent with those of the maximum‐likelihood analysis (Supporting Information Figure S1).

#### 3.2.2 mtCOI gene

After alignment, the fragment had 618 nucleotide sites including 114 parsimony informative sites (111 parsimony informative sites when *Stigmaeopsis longus* was excluded). HG and LW populations each formed a single clade (Figure 5b), as in the phylogenetic network based on parasodium channel gene (Figure 5a). ML populations were split into four clades, again as in the phylogenetic network based on parasodium channel gene sequences (Figure 5). These results were nearly consistent with those of the maximum‐likelihood analysis (Supporting Information Figure S2).

#### 3.2.3 Inference of past demographic history of the three forms

In DIYABC, the highest value of posterior probability was detected for scenario 2 and the value was much higher than the other scenarios, showing no overlapping of 95% hyper probability density with those of the other scenarios (Table 1). For scenario 2, the median values of effective population size of LW, HG, and ML forms (N‐LW, N‐ML, and N‐HG) were 97,500 (95%CI: 31,300–257,000), 419,000 (95%CI: 267,000–496,000), and 180,000 (95%CI: 88,300–285,000), respectively (Table 2a). The median value of *t*1, the divergence time when HG form splits from ML form, was 87,900 (95%CI: 21,600–190,000) generations ago and that of *t*2, the divergence time when LW form splits from ML form, was 320,000 (95%CI: 168,000–395,000) generations ago (Table 2a). The developmental period (from eggs to adults) of the mite under 25℃ is about 17 days for the LW form and 15 days for the HG form, and the estimated low development threshold is 11.7℃ for the LW form and 11.8℃ for the HG form (Saito, Kanazawa, & Sato, 2013). Besides, both form females enter diapause during winter (Saito et al., 2002). Although these life‐history parameters and diapause ability are unknown for the ML form, the minimum and maximum generation numbers can be estimated eight generations and 16 generations per year. Assuming the number of generations per year, the timescales of *t*1 and *t*2 could convert into absolute time as 5,494–10,988 (95%CIs: 1,350–11,875 and 2,700–23,750) years and 20,000–40,000 (95%CIs: 10,500–24,688 and 21,000–49,375) years BP (Table 2b). The median values of the mean mutation rate (per site per generation) and the mean coefficient *k* *C*/*T* were estimated as 8.70 × 10^−^^8^ (95%CI: 6.21 × 10^−^^8^–1.00 × 10^−^^7^) and 1.67 (95%CI: 9.69 × 10^−^^2^–1.10 × 10^−^^1^), respectively. Three of the 39 summary statistics showed significant differences between the observed and simulated data based on the posterior distributions (Supporting Information Table S4). However, the PCA showed that the observed data point was within a small cluster of datasets from the posterior predictive distribution (Supporting Information Figure S3), suggesting that the observed data are generally close to the simulated data, and thus, scenario 2 fits the observed data well.

**Table 2:** Demographic parameters of the scenario 2 obtained by DIYABC (a), and estimated divergence time converted from generation scale to year scale (b)

Parameter	Mean	Median	Mode	Quantile				
				2.5%	5%	95%	97.5%	
(a)								
N‐LW	110,000	**97,500**	75,200	31,300	37,900	230,000	257,000	
N‐ML	409,000	**419,000**	466,000	267,000	291,000	491,000	496,000	
N‐HG	183,000	**180,000**	174,000	88,300	100,000	275,000	285,000	
*t*1	94,400	**87,900**	62,900	21,600	26,800	181,000	190,000	
*t*2	310,000	**320,000**	343,000	168,000	193,000	391,000	395,000	
Mean mutation rate	8.53E−08	**8.70E−08**	1.00E−07	6.21E−08	6.63E−08	9.92E−08	1.00E−07	
Mean coefficient (*k C*/T)	2.72E+00	**1.67E+00**	6.56E−02	9.69E−02	1.58E−01	9.07E+00	1.10E+01	

Time to divergence	Time unit	Mean	Median	Mode	Quantile			
					2.5%	5%	95%	97.5%
(b)								
*t*1	#generation	94,400	**87,900**	62,900	21,600	26,800	181,000	190,000
	#year (min)	5,900	**5,494**	3,931	1,350	1,675	11,313	11,875
	#year (max)	11,800	**10,988**	7,863	2,700	3,350	22,625	23,750
*t*2	#generation	310,000	**320,000**	343,000	168,000	193,000	391,000	395,000
	#year (min)	19,375	**20,000**	21,438	10,500	12,063	24,438	24,688
	#year (max)	38,750	**40,000**	42,875	21,000	24,125	48,875	49,375

Note. N‐form: Effective population sizes of the form. *t*1, *t*2: The number of generations to divergence. For details of scenario 2, see Figure 3. Minimum and maximum numbers of years are estimated by assuming 16 and 8 generations/year. In the text, we mainly use the median values emphasized in bold.

## 4 DISCUSSION

To address the evolutionary process of lethal male fight in the social spider mite, *S. miscanthi*, we investigated male–male aggressiveness in 42 populations and phylogenetic relationships in 47 populations distributed along two land bridges between the Japanese archipelago and the eastern Eurasian continent during the last glacial period. The male weapon morph, an index of male–male aggressiveness, varied among the populations. However, they could be categorized into three forms by the morph, and the presence of the third form with mild aggression (the ML form) was supported besides the LW and HG forms reported in previous papers (Sato, Sabelis, et al., 2013; Sato et al., 2008; Sato, Saito, & Mori, 2000a, 2000b). In the phylogenetic analyses, LW populations and HG populations both clustered into single clades (therefore, the LW form is currently described as a new species, *Stigmaeopsis sabelisi*; Saito, Sato, Chittenden, Lin, & Zhang, 2018). However, ML populations were split into four clades (although one of the four clades distributed in Fuzhou, China, is currently described as a new species, *Stigmaeopsis continentalis*; Saito et al., 2018). The phylogenetic relationships among the clades including the HG and LW forms were unclear.

The ABC approach using mtCOI sequences showed that the most likely scenario is that the ML form in subtropical regions is the ancestral group; the LW form derived from the ML form first, and then, the HG form derived from the ML form (scenario 2 in Figure 3). Although we need to be aware of several sources of uncertainty in the inference such as assumptions about the generation time, overlapping generations, and confidence intervals of the estimated parameters in the ABC (Tsuda, Nakao, Ide, & Tsumura, 2015), the results of the ABC are still highly informative in this study. In the supported scenario, the divergence time of the HG form was estimated to be 5,494–10,988 years (87,900 generations) BP, suggesting that the HG form is quite new and emerged in Japan at the end or after the LGM; that is, a warmer and moister climate returned, and the Japanese archipelago was completely isolated from the Eurasian continent and Taiwan Islands. The divergence time of the LW form was inferred 20,000–40,000 years (320,000 generations) BP. The distribution of the host plant is estimated to have been limited to the coastal areas from southern Taiwan to Hainan close to the South China Sea until 14,000 years BP (Clark et al., 2014). Therefore, the LW form likely emerged before invading the Japanese archipelago, for example, in southern Taiwan and southeastern China. The ranges of divergence times estimated in this study were very broad, especially considering the 95% CI (Table 2). However, the estimated scenario and divergence times correspond well to the expansion history of the host plant estimated in previous study (Clark et al., 2014). This correspondence reinforces our results, as well as those of the study on the host plant (Clark et al., 2014).

The split time of LW and HG forms from the ancestral ML form was estimated, but the time at which the LW form migrated into the Japanese archipelago was not determined in this study. Considering the present geographic and ecological relationships of the LW and HG forms, the LW form possibly had invaded before emergence of the HG form. In eastern Japan, the LW and HG forms are parapatrically distributed with the HG form in the lowlands and the LW form in the highlands (Saito, 1995; Sato, Egas, et al., 2013; Saito & Sahara, 1999; Sato et al., 2008). The LW form may be physiologically able to expand its range into warmer regions (lowland), although the HG form cannot expand its range into colder regions (highland) because of its lighter diapause attribute (Saito et al., 2002). However, the LW form has a difficulty to expand its range into the areas where the HG form occurs, because LW females easily suffer reproductive interference by HG males (Sato et al., 2015, 2008; Sato, Sabelis, et al., 2013). Therefore, if we assume that the LW form occupied Kyushu Islands and Honshu Islands first, subsequently the HG form invaded and drove the LW form into the north and highlands after the climate warmed, then the present geographic and ecological relationship would be easily explained. However, if we assume aerial dispersal in the LW form as reported in some spider mites (Boykin & Campbell, 1984; Lawson, Nyrop, & Dennehy, 1996; Margolies, 1987; Osakabe et al., 2008; Smitley & Kennedy, 1988) and other mites (Bergh & Mccoy, 1997; Duffner, Schruft, & Guggenheim, 2001; Jung & Croft, 2001), any invasion order can cause their parapatric distribution pattern. The possibility of aerial dispersal in the mite may be able to explain why two faunal boundary lines, Osumi strait and Tokara gap, are not important in the mite, different from other animals such as amphibians and reptiles (Ota, 1998). Therefore, for understanding its multiple invasion history, it is important to know its dispersal ability and also gene flow between populations within a form.

The adaptive mechanism of geographic variation in male–male aggressiveness has been addressed from the viewpoint of kin selection theory (Saito, 1995; Sato, Egas, et al., 2013; Saito & Sahara, 1999). We predict that average genetic relatedness in a group is high in LW populations, low in ML populations, and intermediate in HG populations (Sato, Egas, et al., 2013), because of the positive relationship between the likelihood of spring nest establishment by inbreeding and winter harshness (Saito, 1995). According to the kin selection theory in viscous populations (Gardner & West, 2004; Reinhold, 2003), lethal male fight is less favored in LW and ML populations because males encounter only kin males or only nonkin males, whereas lethal male fight is favored in HG populations because males encounter both kin and nonkin males. The results in this study are not against the hypothesis of adaptive mechanism. If the scenarios where the ML form is an admixture of the LW and HG forms (scenario 7 and 8 in Figure 3) were supported as the evolutionary process, the variation in male–male aggressiveness can be generated as a by‐product of the admixture. The scenario supported as the evolutionary process (scenario 2 in Figure 3) suggests that changes in climate, such as winter harshness, can be a key of the divergence in the mite since migration history of the mite during the last glacial period is associated. Difference in climate parameters might affect not only the way of spring nest establishment but also fauna of its natural enemies. Evolution in arthropod herbivores is often associated with natural enemies besides host plants (Schluter, 2000; Tilmon, 2008). Especially, success rate of counterattack against predatory intruders into the nests is low in LW form compared to HG form; that is, male–male aggressiveness is possibly correlated with aggressiveness against predators, although HG form shows similar success rate with *Stigmaeopsis longus *showing no lethal male fight (Saitō, 1986; Yano et al., 2011). Therefore, together with direct test of the kin selection theory by molecular analyses of kin structure, it is also necessary to compare ecology and surrounding environment among the three forms in the mite, especially by revealing those of ML form.

## CONFLICT OF INTEREST

None declared.

## AUTHOR CONTRIBUTIONS

Y.Sato, A.M., and M.E. conceived the ideas; Y.Sato, T.G., Y.Saito, Y‐X.Z., J‐Z.L. J‐T.C., and A.M. collected materials with additional materials from collaborators; Y.Sato, H.S., A.M. Y.Saito, Y‐X.Z., and J‐Z.L. collected the data with assistance from T.G.; Y.T. and Y.Sato analyzed the data; and Y.Sato led the writing with assistance from M.E. and Y.T.

## Data Availability

DNA sequences: Genbank accessions MH015200–MH015213, MH029789–MH029801, MH203500–MH203571.
