# Supplementary material for: Phylogeography of lethal male fighting in a social spider mite
Source: Ecol Evol. 2019 Jan 24;9(4):1590–602. doi: 10.1002/ece3.4770 (PMC6392385; doi:10.1002/ece3.4770)
Supplement: Supplementary file 1 [file ECE3-9-1590-s001.docx]

Supplementary Information Tables S1-S4, and Figures S1-S3

Article title:

Phylogeography of lethal male fighting in a social spider mite

Table S1. Primers used in genetic analyses.

Table S2. Prior distributions of the parameters used in DIYABC.

Table S3. Average relative length of male leg I to leg III in each population used in a cluster analysis of male weapon morph in *Stigmaeopsis miscanthi* (Fig. 4a). For the location of populations, see Fig. 4c.

Table S4. Comparison of summary statistics for the observed data set and posterior simulated data sets in the scenario 2.


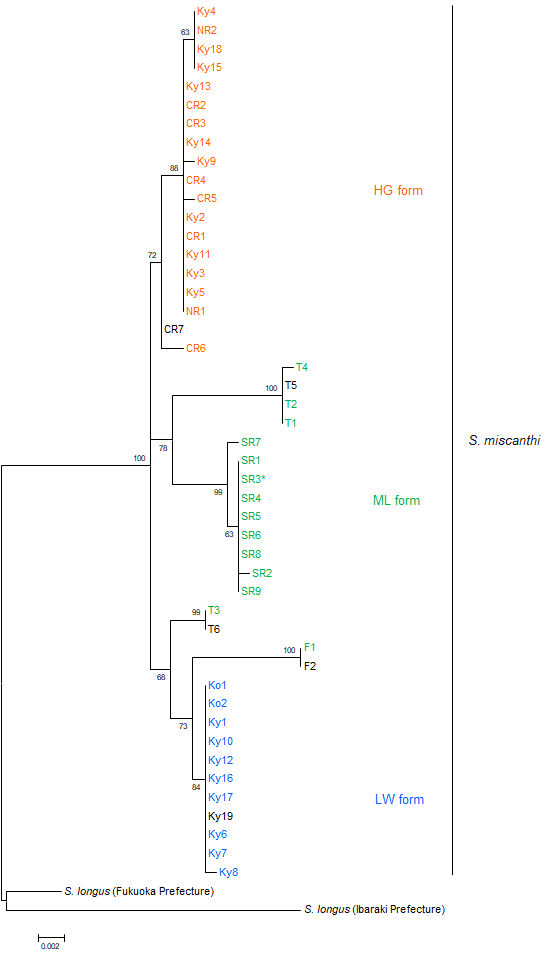


Figure S1. Maximum likelihood phylogenetic tree based on para-sodium channel region of 47 *Stigmaeopsis miscanthi* populations with two *S. longus* populations as the outgroup. Bootstrap values based on 1,000 replicates are shown at the nodes. Orange green and blue populations belong to HG, ML and LW forms respectively according to the male weapon morph (Fig. 4a,b), although green SR3 marked by asterisk was categorized into LW form in the cluster analysis using the male weapon morph (Fig. 4a). For population locations, see Figure 4c.


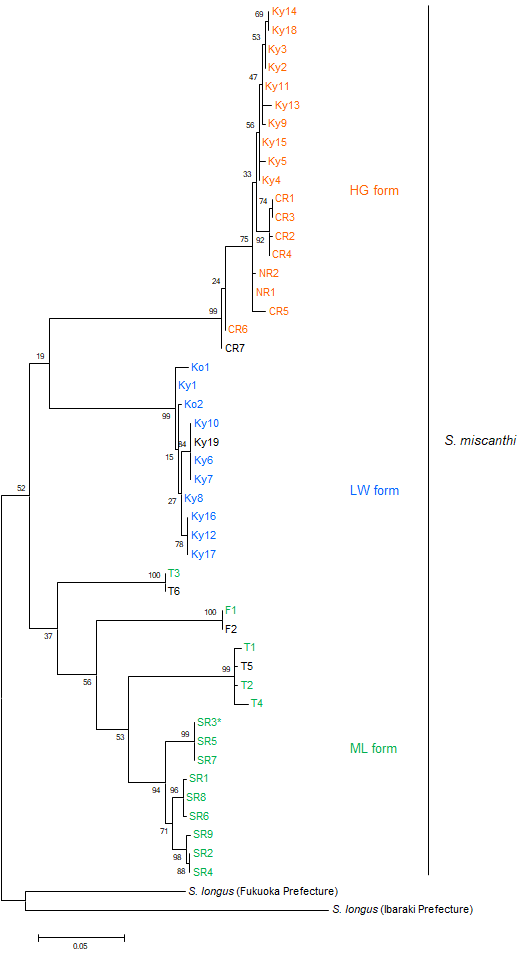


Figure S2. Maximum likelihood phylogenetic tree based on mtCOI of 47 *Stigmaeopsis miscanthi* populations with two *S. longus* populations as the outgroup. Bootstrap values based on 1,000 replicates are shown at the nodes. Orange green and blue populations belong to HG, ML and LW forms respectively according to the male weapon morph (Fig. 4a,b), although green SR3 marked by asterisk was categorized into LW form in the cluster analysis using the male weapon morph (Fig. 4a). For locations of the populations, see Figure 4c.

Figure S3. Principal Component Analysis (PCA) obtained by DIYABC. The observed data set was shown by a circle filled with yellow, the data set of posterior predictive distributions was shown by a circle filled with red, and the data set of prior distributions was shown by a small open red circle.
